# Metal-Free SeBN Ternary-Doped Porous Carbon as Efficient Electrocatalysts for CO_2_ Reduction Reaction

**DOI:** 10.1021/acsaem.2c01201

**Published:** 2022-08-24

**Authors:** Wei Wang, Juan Han, Yan Sun, Miao Zhang, Shiqi Zhou, Kai Zhao, Jiayin Yuan

**Affiliations:** † School of Chemistry and Chemical Engineering, Lanzhou Jiaotong University, Lanzhou 730070, China; ‡ Department of Materials and Environmental Chemistry (MMK), Stockholm University, Stockholm 10691, Sweden

**Keywords:** porous carbon, selenium doping, metal-free electrocatalyst, CO production, CO_2_ reduction reaction

## Abstract

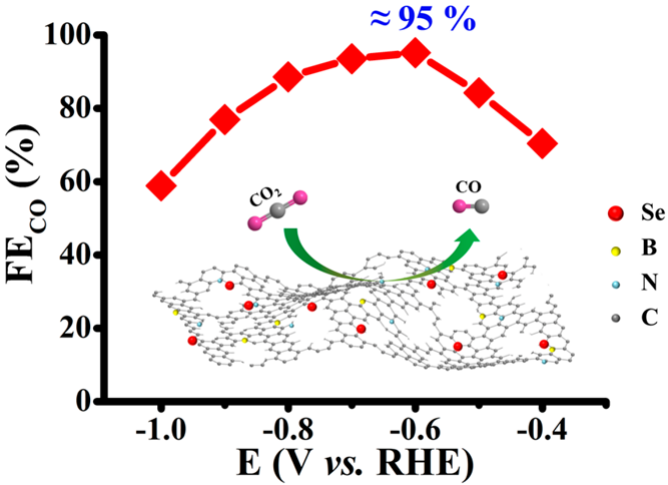

Cost-effective heteroatom-doped porous carbons are considered promising electrocatalysts for CO_2_ reduction reaction (CO_2_RR). Traditionally porous carbons with N doping or N/X codoping (X denotes the second type of heteroatom) have been widely studied, leaving ternary doping a much less studied yet exciting topic to be explored. Herein, a series of electrocatalysts based on metal-free Se, B, and N ternary-doped porous carbons (termed “SeBN-Cs”) were synthesized and tested as metal-free electrocatalysts in CO_2_RR. Our study indicates that the major product of CO_2_RR on the SeBN-C electrocatalysts was CO with a small fraction (<5%) of H_2_ as the byproduct. The optimal electrocatalyst sample SeBN-C-1100 prepared at 1100 °C exhibits a high CO selectivity with a Faradaic efficiency of CO reaching 95.2%. After 10 h of continuous electrolysis operation, the Faradaic efficiency and the current density are maintained high at 97.6 and 84.7% of the initial values, respectively, indicative of a long-term operational stability. This study provides an excellent reference to deepen our understanding of the properties and functions of multi-heteroatom-doped porous carbon electrocatalysts in CO_2_RR.

## 1 Introduction

The steadily rising concentration of CO_2_ in the atmosphere has been one of the most significant global challenges of our times because it is considered to be largely responsible for global warming, climate change, ocean acidification, and related environmental disasters, e.g., local extreme weather. To mitigate this issue, tremendous efforts have been taken globally, and CO_2_ capture, storage, and utilization (CCSU) is regarded as a promising solution.^1−4^ In terms of CO_2_ utilization, electrocatalytic CO_2_ reduction reaction (CO_2_RR) into valuable fuel and chemical feedstock carries a bright prospect due to its mild reaction temperature and pressure, which can be advantageously driven by renewable electricity to achieve a negative net emission of CO_2_.^5−7^ Generally speaking, assisted by tailor-made electrocatalysts, the CO_2_RR can target a wide range of hydrocarbon products, including CO, CH_4_, HCOOH, and more; the competing side-reaction produces H_2_ via the hydrogen evolution reaction (HER). In this context, the low product selectivity remains one of the major problems that retards CO_2_RR technology for practical large-scale implementation. The rational design and synthesis of the electrocatalyst, as a key factor to govern the product composition, is of paramount significance.^8,9^

Recently, the employment of transition-metal-based (e.g., Pd, Au and Zn) electrocatalysts in CO_2_RR has been extensively studied because of the very active metal surface capable of catalytic CO_2_ conversion. Despite much improvement in catalytic activity, many metal-containing electrocatalysts suffer from a high cost and/or a low product selectivity, not to mention the profound side-reaction due to HER.^10,11^ In this regard, metal-free heteroatom-doped carbonaceous electrocatalyst has been receiving much interest due to their wide accessibility, rich natural resources, high surface area, adjustable pore structure, sufficient thermal and chemical stability (e.g., under strong acidic and alkali conditions), being free of metal-leaching, and environmental friendliness.^12,13^ Normally, the chemical bonding of heteroatoms into the carbon framework can modulate, and if designed well, significantly promote its catalytic activity due to a possible accurate control of their surface properties and/or bulk electronic structures.^14^ Considering previous studies, N has been so far the most frequently studied heteroatom for doping carbon because of its broad existence in many organic materials and its atomic size analogous to the carbon atom that makes N atoms easily bonded covalently into the carbon matrix. At present, N single-doped carbons have been extensively studied in particular with respect to their electrochemical catalytic performance. Along this line, N/X binary doping, where X stands mostly for B, S, and P, and atomically dispersed metals as well as semimetals have also been reported to upgrade the carbocatalyst for CO_2_RR.^15^ It is expected that doping carbons with multiple heteroatoms may largely broaden the property and function window of the carbonaceous electrocatalysts for target electrochemical reactions.^16^ By contrast to the prosperous activities in mono- or binary heteroatom doping, the ternary doping by three different types of heteroatoms has been rarely reported due to a higher level of structural complexity.^16−18^

While functionalization of carbons with nonmetals in a form of covalent bonds and with metals in a common form of single atoms is popular, there have been limited case studies on the semimetal doping into a carbon network. The semimetal Se, an element in the chalcogen group, possesses an electronegativity of 2.55 almost equivalent to C (2.58) but a higher polarizability, a larger atomic size, and more abundant d-orbital electrons that can be partially transferred to the carbon network. In addition, Se doping can introduce extra catalytically active centers to tune the charge redistribution among carbon atoms, which are beneficial to tailor the electrical transport property of a carbon skeleton.^19^ Furthermore, some promising but very preliminary studies showed that Se-doped carbon materials are electrochemically active, e.g., in water splitting and hydrogen production,^20,21^ where the study of CO_2_RR is scarce.^22^ It is noteworthy that the introduction of Se atoms together with other heteroatoms (e.g., N) simultaneously into the carbon skeleton may adjust the charge distribution both on the pristine carbon surface and in the bulk in a broad scope, as the large-sized Se atoms are believed to be bonded preferentially on the rim of the carbon matrix to modulate the surface properties of carbon frameworks, and the N is more effective in changing the bulk properties of carbon due to its similar atomic size to carbon. Thereby, simultaneous doping of carbons with Se and other elements may open up more structural possibilities for electrocatalysis.^23,24^

Herein, a series of metal-free, Se, B, and N ternary-doped porous carbon electrocatalysts (termed “SeBN-C-*x*” thereafter, where *x* denotes the carbonization temperature) were fabricated by a facile pyrolysis method. By carefully optimizing the carbonization temperature and the dopant content, the best-performing SeBN-C-1100 electrocatalyst was identified that demonstrated a superior performance in CO_2_RR. Concretely speaking, the SeBN-C-1100 reached a CO Faradaic efficiency (FE_CO_) of as high as 95.2%. After a 10 h continuous CO_2_RR operation, the FE_CO_ and the current density maintained the initial performance by 97.6% and 84.7%, respectively, proving a long-term operational stability in practical use.

## 2 Experimental Section

### 2.1 Preparation of the SeBN-C Electrocatalysts

**Figure 1:**
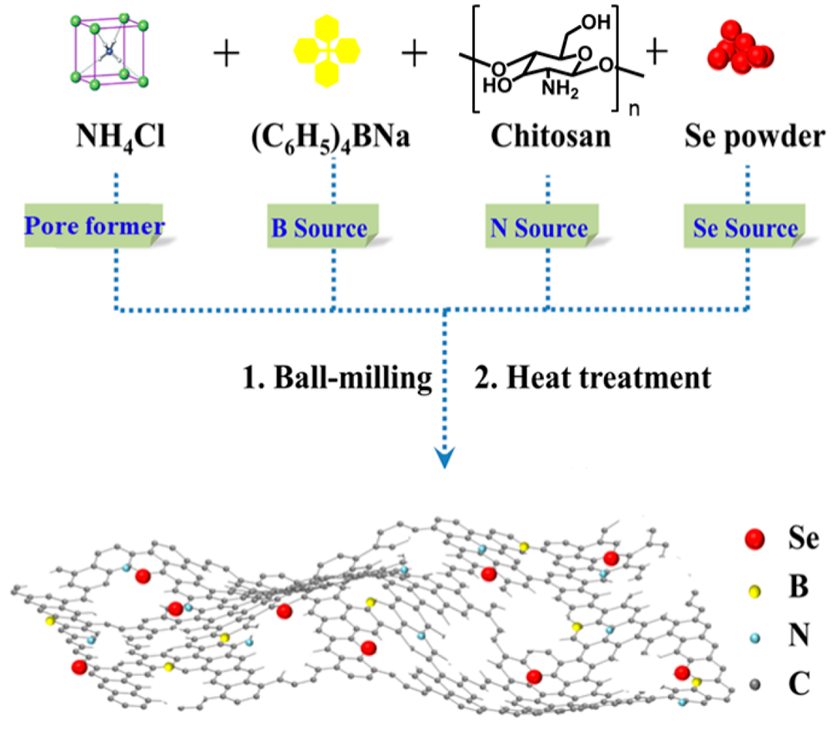
Schematic illustration of the synthetic route to the SeBN-C-*x* electrocatalysts.

In detail, a series of SeBN-C electrocatalysts were developed in a strategy shown in Figure 1. First, chitosan as a source of C and N, Se powder for Se, (C_6_H_5_)_4_BNa for B, and NH_4_Cl as a pore maker were uniformly mixed and homogenized microscopically by ball-milling treatment. Then, the mixture was transferred to a tube furnace for prepyrolysis treatment at 300 °C for 1 h under Ar saturation and next heated to the final temperature at a heating rate of 5 °C min^–1^. It was kept at the final temperature for 2 h under the same situation. The final temperature in the second heat treatment was set at 900, 950, 1000, 1050, 1100, or 1150 °C. The corresponding products are termed as “SeBN-C-*x*”, where *x* denotes the final carbonization temperature. In a similar preparation procedure as SeBN-C-*x*, the three reference samples SeN-C, BN-C, and N-C were prepared, in which (C_6_H_5_)_4_BNa, Se powder, and both (C_6_H_5_)_4_BNa and Se powder were absent in the carbonization process, respectively. The final carbonization temperature was set at 1100 °C for the three reference samples, as it was an optimal temperature found for the SeBN-C-*x* samples. To note that during the pyrolysis at a temperature above 338 °C, i.e., the sublimation temperature of NH_4_Cl, NH_4_Cl started simultaneously to decompose into NH_3_ and HCl to release a large amount of gas molecules and thereby generated rich voids inside the mixture. For this sake, a fairly macroporous structure was obtained in the product that can facilitate the reactants in the electrolyte to come in better contact with the electrocatalysts.

### 2.2 Measurements

The CO_2_RR electrochemical tests were performed on a CHI 660E electrochemical workstation in a three-electrode system at room temperature. It was conducted in an H-type cell. In a typical run, 5.0 mg of electrocatalyst was sonically dispersed in a mixture of ethanol and Nafion (5 wt % in isopropanol solution) to prepare the electrocatalyst ink. Then, 200.0 μL of electrocatalyst ink was dropped onto a carbon paper electrode (1 cm × 1 cm) and dried subsequently. The gaseous quantity of products was determined online by gas chromatography (GC; FL9790II; standard curves in Figure S1). The liquid products were quantitatively analyzed by a nuclear magnetic resonance (NMR) spectrometer (Bruker Avance III 500 MHz).

Transmission electron microscopy (TEM) and energy-dispersive X-ray spectroscopy (EDX) were performed on an FEI TECNAI G2 TF20 S-TWIN TMP microscope. X-ray powder diffraction (XRD) measurements were performed on a Rigaku 92 D/Max-2400 diffractometer. Raman spectra were operated with a Bruker RFS100/S spectrometer. X-ray photoelectron spectroscopy (XPS) data were obtained on a Kratos Axis Ultra DLD spectrometer. The specific surface area and pore size distribution were determined by using an accelerated surface area and porosimetry (ASAP) 2020 system. Reagents, characterization methods, and more details are listed in the Supporting Information.

## 3 Results and Discussion

### 3.1 Electrocatalytic Performances

Compared with metal-containing electrocatalysts, metal-free carbocatalysts have the merits of rich natural abundance, low price, broad accessibility, good chemical tolerance in acidic or basic environments, and low toxicity. Moreover, their catalytic function can be readily modulated by various approaches, among which, the introduction of heteroatoms of different types and amounts receives increasing interest. The semimetal chalcogen element Se draws our interest, as several previous studies have demonstrated its dopant role in enhancing carbon’s electrical transport performance.^16^ Considering the multi-heteroatom doping effect that may synergistically promote electrocatalytic activity, as reported in the literature,^25,26^ we were motivated to move forward the research frontline by investigating the Se-containing multi-heteroatom-doped carbon electrocatalyst, here the SeBN-C-*x* samples.

**Figure 2:**
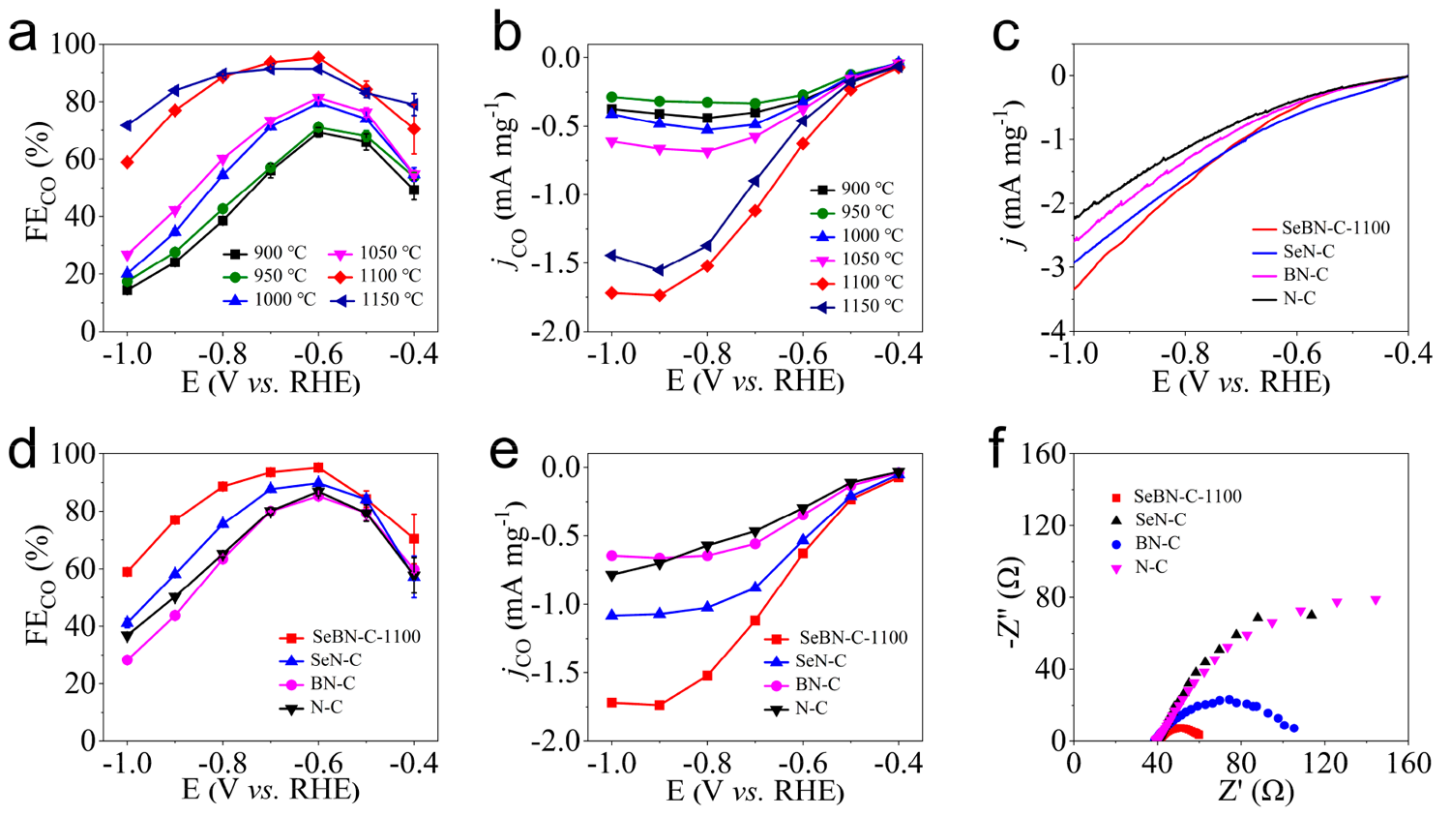
Electrocatalytic performances of SeBN-C-*x* catalysts (*x* denotes the final carbonization temperature). (a, b) FE_CO_ and *j*_CO_ plots of CO_2_RR on SeBN-C-*x* (*x* = 900, 950, 1000, 1050, 1100, and 1150 °C), respectively. (c–e) LSV, FE_CO_, and *j*_CO_ plots, respectively, of SeBN-C-1100, SeN-C, BN-C, and N-C in CO_2_-saturated 0.1 M KHCO_3_ solution. (f) Nyquist plots of SeBN-C-1100, SeN-C, BN-C, and N-C in N_2_-saturated 0.1 M KHCO_3_ solution.

Because the final-step carbonization temperature is one of the foremost factors affecting the CO_2_RR performance of our carbocatalysts, the linear sweep voltammetry (LSV), Faradaic efficiency (FE), and the partial current density (*j*) of SeBN-C-*x* electrocatalysts prepared at a temperature from 900 to 1150 °C were studied first. As shown in Figure 2a, the FE_CO_ values of all SeBN-C-*x* samples (*x* = 900, 950, 1000, 1050, 1100, and 1150 °C) show an overall similar trend, starting at −0.4 V with 49.3, 53.9, 54.7, 54.8, 70.4, and 79.0%, respectively, and reaching a maximum FE_CO_ value of 69.3, 71.0, 79.5, 81.4, 95.2, and 91.3% at −0.6 V, accordingly. Next, they gradually decreased at <−0.6 V and ended up with a final FE_CO_ value of 14.4, 17.3, 20.1, 26.8, 58.9, and 71.7%, respectively, at −1.0 V. To be highlighted here is the SeBN-C-1100, which reaches the largest FE_CO_ of 95.2% at −0.6 V in Figure 2a. Meanwhile in the LSV curves (Figure S2), from −0.4 to −1.0 V, the partial current density of CO (*j*_CO_) becomes more negative. Here, the high reduction current density value is favored as it is expectedly more conducive to the CO_2_RR process.

Because HER is a competitive reaction of CO_2_RR, the Faradaic efficiency of H_2_ (FE_H2_) was found in an opposite trend to FE_CO_. FE_H2_ of all samples decreases first from −0.4 to −0.6 V and then increases from −0.6 to −1.0 V, leaving a minimum peak at −0.6 V (Figure S3a). Additionally, the *j*_CO_ of SeBN-C-*x* samples is shown in Figure 2b. All appear in a similar trend to the FE_CO_ plot along with the carbonization temperature in the potential range from −0.4 to −1.0 V. Concretely speaking, starting from −0.4 V, the *j*_CO_ values of all SeBN-C-*x* samples sequentially increase to a maximum value of −0.44, −0.33, −0.53, −0.68, −1.74, and −1.55 mA mg^–1^ at −0.8, −0.7, −0.8, −0.8, −0.9, and −0.9 V, respectively, and then gradually decrease until −1.0 V. Because the more negative potential is favorable to HER, unlike *j*_CO_, the partial current density of H_2_ (*j*_H_2__) of all six SeBN-C-*x* samples continues to increase from −0.4 to −1.0 V (Figure S3b). These results altogether indicate that the SeBN-C-1100 sample has the best CO_2_RR catalytic performance of its highest FE_CO_ and largest *j*_CO_ value. This is presumably attributed to the combination of a high conductivity and a suitable doping content of heteroatoms. According to a previous study,^27^ a higher carbonization temperature caused a higher degree of graphitization and thereby a better conductivity to favor its CO_2_RR. However, the heteroatoms, in particular, the N species that serves as the main catalytic active sites for CO_2_RR, will reduce their doping content at a higher carbonization temperature.^28^ Thus, the SeBN-C-1100 is expected to reach a good balance between these two factors, i.e., the graphitization degree and the content of heteroatom doping.

To generate a deeper insight into the CO_2_RR performance of SeBN ternary-doped samples, a set of comprehensive electrochemical tests were conducted to SeBN-C-1100 as the optimal sample and the three references, i.e., SeN-C (without B doping), BN-C (without Se doping), and N-C (without B and Se doping), that were prepared at 1100 °C as well. As seen in the LSV curve in Figure 2c, the current density drops in all of these samples when shifting the potential from −0.4 to −1.0 V, reaching −3.40, −2.99, −2.65, and −2.32 mA mg^–1^ for SeBN-C-1100, SeN-C, BN-C, and N-C, respectively. It is clear that in comparison with SeN-C, BN-C, and N-C, the sample of SeBN-C-1100 has a sharper drop and reaches the lowest minimum current density of −3.40 mA mg^–1^ among all tested samples. Furthermore, the FE_CO_ plot in Figure 2d verifies this statement as well. The FE_CO_ of SeBN-C-1100, SeN-C, BN-C, and N-C varies in an analogous trend, first rising from 70.4, 57.1, 59.8, and 57.7% at −0.4 V to the maximum value of 95.2, 89.8, 85.3, and 86.9% at −0.6 V, and then gradually decreasing to 58.9, 41.1, 28.2, and 36.9% at −1.0 V, respectively. Contrary to FE_CO_, the FE_H_2__ of SeBN-C-1100, SeN-C, BN-C, and N-C peaked with the minimum value of 4.8, 10.2, 14.7, and 13.1% at −0.6 V, respectively, and then increased no matter when the potential changed from −0.6 V to −0.4 V or to −1.0 V (Figure S4a).

The *j*_CO_ values of all electrocatalysts were further studied as shown in Figure 2e. The SeBN-C-1100 and BN-C have the same variation trend, i.e., first increasing to the maximum values of −1.74 and −0.66 mA mg^–1^ at −0.9 V, respectively, and then decreasing gradually to −1.0 V. This behavior can be attributed to the rapid consumption of CO_2_ at a low solubility or slow mass transfer under a high CO production rate.^8^ Unlike the former two samples discussed, the *j*_CO_ of SeN-C and N-C continues to increase over the entire potential range from −0.4 to −1.0 V, until it reaches the maximum values of −1.08 and −0.78 mA mg^–1^ at −1.0 V, respectively. It is worth noting that in comparison to SeN-C, BN-C, and N-C, the as-prepared SeBN-C-1100 exhibits the largest *j*_CO_ in the overall potential range. Meanwhile, the *j*_H_2__ of all electrocatalysts continues to increase over the entire potential range, as the more negative the potential is the more favored the HER (Figure S4b). In addition, among all tested electrocatalysts, the SeBN-C-1100 presents the highest current density and favorable durability at all applied potentials in the CO_2_RR process, as supported by the testing results (Figure S5). Advantageously, we found that CO and H_2_ are the only detected products and no liquid-phase product was spotted after electrolysis in the cathode compartment electrolyte by ^1^H NMR (Figure S6). As shown in Figure 2f, among all samples, the SeBN-C-1100 has the lowest interface charge transfer resistance and thus the optimized conductivity. All these results point out that Se, B, and N ternary doping facilitates the Faraday process and promotes the kinetics of CO_2_ activation.

According to the aforementioned analysis, it can be inferred that the Se, B, and N ternary-doped carbon electrocatalyst has the best catalytic activity in the CO_2_RR studied here. There seems to be a synergistic effect of the Se, B, and N codoping. Among various effects, we assume that the ternary doping above all can redistribute the surface charge of the electrocatalyst, which enables the positively charged carbon atoms to promote the adsorption of CO_2_.^29,30^ In addition, the relatively high carbonization temperature at 1100 °C enhances the electron delocalization and improves the conductivity.^31,32^ Moreover, the large-sized Se atoms in the carbon framework will favorably introduce more structure defects, as their large size will restrict the dense packing of the adjacent graphitic layers so to amplify the diffusion kinetics through the graphitic layers.

**Figure 3:**
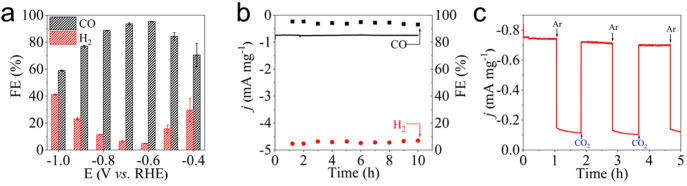
(a) Plot of the FE vs the applied potential for SeBN-C-1100. (b) Stability test of electrocatalytic CO_2_RR on the SeBN-C-1100 at −0.6 V. (c) Current density vs time plot on the SeBN-C-1100 between alternating Ar- and CO_2_-saturated 0.1 M KHCO_3_ solution at −0.6 V.

To fully evaluate the CO_2_RR performance of SeBN-C-1100, in-depth electrochemical characterization was carried out. Compared with the Ar-saturated electrolyte, the LSV curve of SeBN-C-1100 shows a more positive onset potential of −0.48 mA mg^–1^ at −0.6 V under the CO_2_-saturation condition, which supports that it has good electrocatalytic activity in CO_2_RR (Figure S7). As displayed in Figure 3a, the FE_CO_ of SeBN-C-1100 is 70.4% at −0.4 V and then gradually increases with a negative shift of the potential, reaching a maximum value of 95.2% at −0.6 V, before it decreases then to 58.9% at −1.0 V. Because CO_2_RR and HER are competing reactions, the corresponding FE_H2_ decreased from 29.6% at −0.4 V to the minimum value of 4.8% at −0.6 V and then slowly increased to 41.1% at −1.0 V. At the same time, in comparison with other metal-free carbon electrocatalysts to reduce CO_2_ to CO in previous reports, the SeBN-C-1100 possesses a fairly high FE_CO_ (Table S1). Additionally, durability was also investigated as it is one of the key indicators for practical use. In Figure 3b, the current density attenuation during the 10 h continuous electrolysis process is negligible, and the FE_CO_ and FE_H2_ of SeBN-C-1100 practically maintained constant at 94.5 and 4.5%, respectively. After the 10 h test, the FE_CO_ of SeBN-C-1100 only lost 2.4%, and the current density maintained 84.7% of the initial value, indicating that SeBN-C-1100 has satisfactory durability for a long-time operation.

Because the source of CO_2_ in the electrocatalytic CO_2_RR process was controversial in the literature, a control experiment of Ar- and CO_2_-saturated electrolyte was carried out. As shown in Figure 3c, when the electrolyte is saturated by Ar, the current density is −0.11 mA mg^–1^, while a much higher value of −0.73 mA mg^–1^ was received in the CO_2_-saturated electrolyte. This outcome implies that the electrolyzed CO_2_ comes indeed from the dissolved CO_2_ in the electrolyte, rather than the preadsorbed CO_2_ before the electrocatalytic experiment. At the same time, the FE of the corresponding reduction products and the results of flame ionization detection (FID) and thermal conductivity detector (TCD) on gas chromatography also support this conclusion (Figure S8). Under a CO_2_-saturated situation, only CO was detected in the FID. By contrast, under Ar saturation, only H_2_ was detected in the TCD.

### 3.2 Physical Property Characterizations

**Figure 4:**
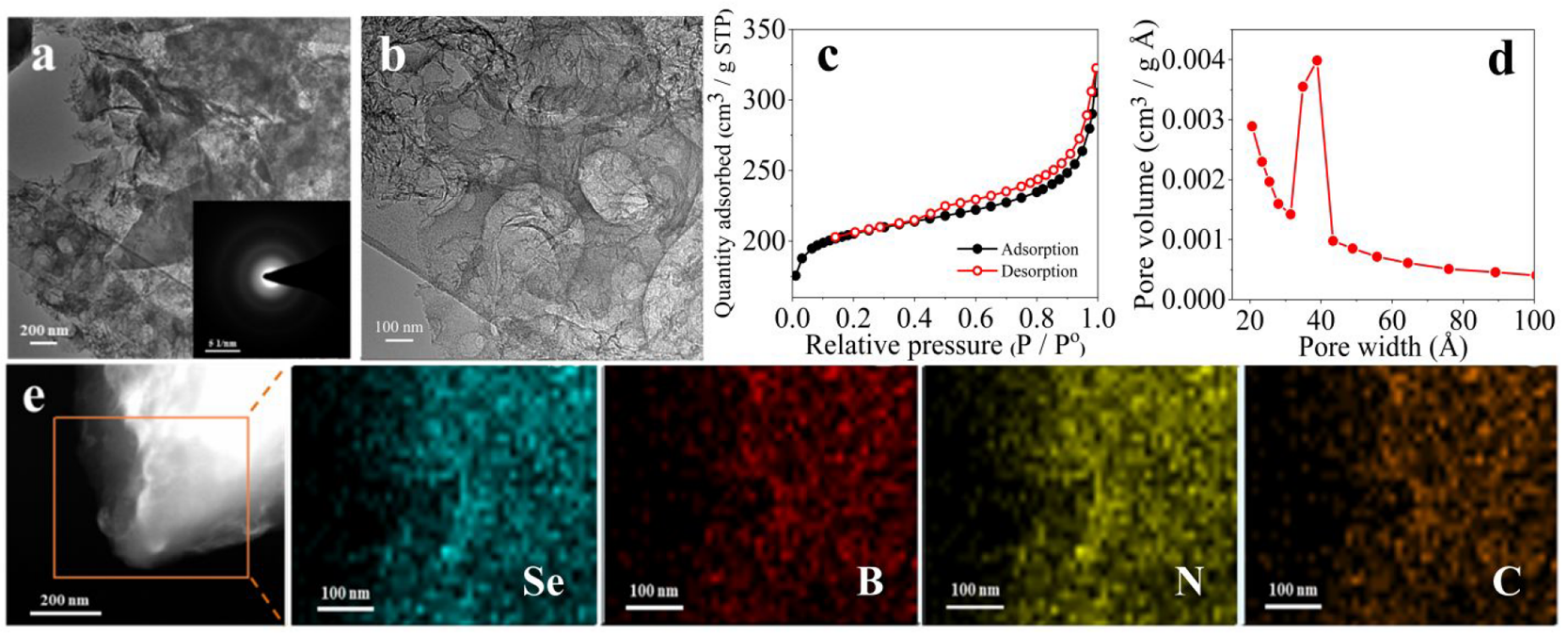
(a, b) Representative TEM images of SeBN-C-1100. The inset in (a) is the corresponding SAED pattern. (c) N_2_ adsorption–desorption isotherms of SeBN-C-1100. (d) The corresponding pore size distribution diagram. (e) Elemental mapping (Se, B, N, and C) images of SeBN-C-1100.

The transmission electron microscopy (TEM) images of SeBN-C-1100 (Figure 4a,b) display its microscopic morphology at low and high resolutions. It can be seen that SeBN-C-1100 exhibits a wrinkled two-dimensional layered texture, where macropores are spotted directly in the layers. The TEM images of SeN-C (Figure S9), BN-C (Figure S10), and N-C (Figure S11) appear similar to the SeBN-C-1100 sample. The two-dimensional layered carbon itself has a larger surface area than normal particulate carbon powders with a less exposed surface; the existence of the macroporous structure as observed in the TEM images further expands the pore volume and reduces diffusion resistance. This makes active sites more accessible for the catalytic reaction and at the same time promotes the electrolyte to better contact the active sites, thereby speeding up the CO_2_RR kinetics.^33,34^ In addition, the corresponding selected area electron diffraction (SAED) patterns of SeBN-C-1100, SeN-C, BN-C, and N-C electrocatalysts (insets in Figures 4a, S9a, S10a, and S11a, respectively) all exhibit a typical polycrystalline style that can be assigned to graphitic carbons.

The N_2_ adsorption–desorption isotherms of SeBN-C-1100 in Figure 4c exhibit a type-IV-like character. The weak but still detectable hysteresis loop corresponding to the capillary filling of porous carbons appears in the middle partial pressure section. In Figure 4d, the pore size distribution plot of SeBN-C-1100 clearly displays a dominant mesopore size at 3.4–3.9 nm. The surface area of SeBN-C electrocatalyst calculated by the Brunauer–Emmett–Teller (BET) equation is 691 m^2^ g^–1^. This large specific surface area provided by the hierarchically macro-/mesoporous structure with a uniform sub-10 nm mesopore size is beneficial to the electrocatalytic CO_2_RR.^35^ The element mapping images in Figure 4e reflect the homogeneous distribution of Se, B, N, and C elements in SeBN-C-1100 at a nanoscopic scale, indicating that the three heteroatoms have been successfully introduced evenly into the carbon matrix. The energy-dispersive X-ray spectroscopy (EDX) image of SeBN-C-1100 further confirms the existence and relative intensities of Se, B, N, and C elements (Figure S12).

**Figure 5:**
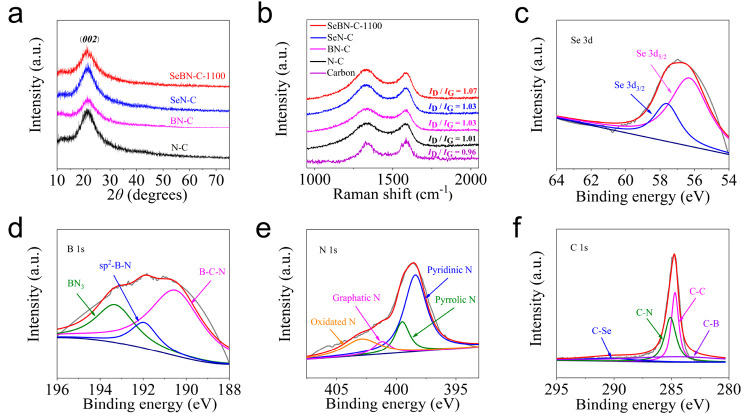
(a) XRD patterns of SeBN-C-1100, SeN-C, BN-C, and N-C. (b) Raman spectra of SeBN-C-1100, SeN-C, BN-C, N-C, and carbon. (c–f) High-resolution XPS spectra of Se 3d (c), B 1s (d), N 1s (e), and C 1s (f).

To study the doping effect on the defects and phase structure, the X-ray diffraction (XRD) and Raman spectroscopy of SeBN-C-1100, SeN-C, BN-C, and N-C were tested. As shown in Figure 5a, the black curve with a diffraction peak centered at 21.2° corresponds to the (*002*) plane of the graphitic carbon on sample N-C. After Se and B are introduced separately or jointly, there is no obvious change in the peak position, indicating that the introduction of these heteroatoms has little or no effect on the carbon phase structure. Raman spectroscopy is commonly used to evaluate the crystalline structure and defects of electrocatalytic materials.^36^ As shown in Figure 5b, the two peaks at 1329 and 1586 cm^–1^ correspond to the structural defects and disorder (D band) and the graphitic sp^2^ carbon (G band), respectively. In addition, the *I*_D_/*I*_G_ value is usually employed to evaluate the density of defects in carbon materials,^37^ and its value is calculated from the peak intensities of the D band and G band. Compared with the Raman spectra of pure carbon powders, the positions of the D and G bands of SeBN-C-1100, SeN-C, BN-C, and N-C are all shifted due to the incorporation of heteroatoms into the carbon skeleton. Meanwhile, SeBN-C-1100 (1.07), SeN-C (1.03), BN-C (1.03), and N-C (1.01) exhibited a higher *I*_D_/*I*_G_ value, thus a lower degree of graphitization than C (0.96), implying that doped heteroatoms serve as and/or induce disordered sites that are potentially useful in catalysis. It is worth noting that analysis of the Raman spectra here is based on the model built up for nondoped carbons; thus, the true meaning behind these values might be subject to further discussion. Also, it can be seen that SeBN-C-1100 has a slightly higher *I*_D_/*I*_G_ value for SeBN-C-1100 (1.07) than SeN-C (1.03), BN-C (1.03), and N-C (1.01), respectively, which implies that the simultaneous introduction of Se, B, and N heteroatoms indeed introduces more structural defects. Besides, Raman characterization was performed for the SeBN-C samples prepared at different temperatures (Figure S13). The SeBN-C-1100 is slightly higher or similar to the *I*_D_/*I*_G_ values of 900 °C (1.06), 950 °C (1.07), 1000 °C (1.06), and 1050 °C (1.05) samples, which may be caused by more structural defects and exposed edge planes. However, its *I*_D_/*I*_G_ value of SeBN-C-1100 is obviously lower than SeBN-C-1150 (1.09), indicating that the defect density may be slightly inferior to SeBN-C-1150 sample.

To study the oxidation state of Se, B, N, and C, X-ray photoelectron spectroscopy (XPS) analysis was further carried out. The XPS survey of SeBN-C-1100 shows five peaks that correspond to Se 3d (56.8 eV), B 1s (183.8 eV), C 1s (284.8 eV), N 1s (399.2 eV), and O 1s (532.5 eV), respectively (Figure S14). The Se, B, and N atoms account for 0.3, 1.5, and 2.6% in the electrocatalyst, respectively (Figure S15). In a close-up view, the high-resolution XPS spectrum of Se 3d in Figure 5c shows two adjacent peaks at 56.3 and 57.6 eV, corresponding to the spin–orbit splitting of Se 3d_5/2_ and Se 3d_3/2_, respectively.^38^ Meanwhile, the high-resolution XPS spectrum of B 1s (Figure 5d) shows three peaks at 190.5, 192.0, and 193.3 eV. These three peaks coincide with its three possible atomic structures, i.e., B–C–N, sp^2^-BN, and BN_3_, respectively, confirming that B has been doped atomically into the carbon matrix in different chemical environments.^39^ Moreover, the N 1s spectrum (Figure 5e) shows that SeBN-C-1100 contains four N states, i.e., pyridinic N (398.4 eV), pyrrolic N (399.5 eV), graphitic N (401.2 eV), and oxidated N (402.9 eV).^40^ In Figure 5f, the high-resolution XPS spectrum of C 1s shows expectedly four peaks, corresponding to C–B (284.5 eV), C–C (284.8 eV), C–N (285.1 eV), and C–Se (290.4 eV).^41−43^

According to the above discussion, the excellent electrocatalytic performance of our metal-free SeBN-C-1100 catalyst can be presumably attributed to the following aspects: (i) Se’s high polarizability can enhance electron transport through the carbon skeleton and provide more active sites in CO_2_RR.^16^ (ii) These ternary-doped carbon materials that simultaneously introduce Se, B, and N heteroatoms of different electronegativity can produce a structural synergy between the heteroatoms. Individually, Se atoms with semimetallic properties may lead to covalent edge doping, and the electron-deficient B atoms and electron-rich N atoms may lead to p-type and n-type doping, respectively, which offers a wide window to tune the physical and chemical properties of the porous carbon materials.^16^ Not only the bulk but also the surface charge distribution on carbon is expected to be regulated by the three distinctive heteroatoms to build up more active sites. (iii) The optimal SeBN-C-1100 electrocatalyst has a sheet-like structure, which assists the ion transport and an enhanced contact between the reactants in the electrolyte and the electrocatalyst. All in all, the SeBN-C-1100 prepared from low-cost chemicals in a simple fabrication step is a cost-effective, scalable option for producing CO through CO_2_RR. It would provide a valuable reference for future preparation and study of multi-heteroatom-doped carbon electrocatalysts.

## 4 Conclusions

In conclusion, a series of metal-free porous SeBN-ternary doped porous carbons as electrocatalysts were prepared. The introduced Se, B, and N heteroatoms into the porous carbon network at 1100 °C offer the best electrocatalytic CO_2_RR activity in the production of CO at a FE_CO_ = 95.2% at −0.6 V. After a continuous 10 h electrolysis operation, the FE_CO_ and current density were maintained at 97.6 and 84.7% of the initial values, respectively, supporting its satisfactory durability. This SeBN-C electrocatalyst system has promising application prospects in catalyzing CO_2_RR and will provide a valuable example for Se-containing multi-heteroatom-doped carbon electrocatalysts in a future study.

## Supporting Information Available

Physical characterization data; details of electrochemical tests and analysis of CO_2_ reduction products; gas chromatography standard curves; LSV curves; FE_H_2__and *j*_H_2__ on SeBN-C-*x* (900–1150 °C); FE_H_2__ and *j*_H_2__ plots of CO_2_RR; current density variation during CO_2_RR at various potentials; ^1^H NMR analysis data after CO_2_RR operation; LSV curves under Ar and CO_2_-saturation; analysis of reduction products on FID and TCD detectors; TEM images of SeN-C, BN-C, and N-C; EDX analysis of SeBN-C-1100; Raman spectra; full XPS spectrum and elemental content of SeBN-C-1100; comparison with literature reports (PDF)
