# Supplementary material for: Metal-Free SeBN Ternary-Doped Porous Carbon as Efficient Electrocatalysts for CO2 Reduction Reaction
Source: ACS Appl Energy Mater. 2022 Aug 24;5(9):10518–25. doi: 10.1021/acsaem.2c01201 (PMC9516553; doi:10.1021/acsaem.2c01201)
Supplement: Supplementary file 1 — ae2c01201_si_001.pdf [file ae2c01201_si_001.pdf]

Supporting Information

Metal-free SeBN ternary-doped porous carbon as efficient electrocatalyst for CO_2 reduction reaction

Wei Wang[a,*] Juan Han[a], Yan Sun[a], Miao Zhang[b], Shiqi Zhou,[b] Kai Zhao[a], Jiayin Yuan[b,*]

[a]*School of Chemistry and Chemical Engineering, Lanzhou Jiaotong University, Lanzhou 730070, China*

[b]*Department of Materials and Environmental Chemistry (MMK), Stockholm University, Stockholm 10691, Sweden*

Corresponding Authors

Wei Wang, Email: wangw@mail.lzjtu.cn (W. Wang)

Jiayin Yuan, Email: jiayin.yuan@mmk.su.se (J. Yuan)

1. Materials

Chitosan (BR: Biological Reagent) is obtained from Shanghai Jingchun Reagent Co., Ltd. Se powder (AR: Analytical Reagent) and $(C_6H_5)_4BNa$ (AR) are acquired from Shanghai Zhongqin Chemical Reagent Co., Ltd. NH_4Cl (AR) is obtained from Tianjin BASF Chemical Co., Ltd. Nafion solution (AR) was purchased from Dupont (USA). $KHCO_3$ (AR) was acquired from Shanghai Energy Chemical Co. Ethanol (AR) was obtained from Shanghai Aladdin Biochemical Technology Co. Ltd. All reagents were used without further purification. Ar (> 99.999 %) and CO_2 (> 99.999 %) were acquired from Lanzhou Yulong Gas Inc.

2. Characterizations

Transmission electron microscopy (TEM), and energy-dispersive X-ray spectroscopy (EDX) was performed on FEI TECNAI G2 TF20 S-TWIN TMP microscope (USA). X-ray powder diffraction (XRD) measurements were carried out on a Rigaku 92 D/Max-2400 diffractometer (Japan) employing Cu $K\alpha$ radiation at 40 kV and 150 mA (scan rate: 10° min^{-1}). Raman spectra were operated with a Bruker RFS100/S spectrometer (Germany; laser wavelength: 663.8 nm; spot size: ~1μm; laser power: ~10 mW). X-ray photoelectron spectroscopy (XPS) data were obtained on a Kratos Axis Ultra DLD spectrometer (Japan) using radiation source Al $K\alpha$ with an energy of 1486.6 eV. The specific surface area and pore size distribution were determined using an accelerated surface area and porosimetry (ASAP) 2020 system. The pretreatment conditions for the gas sorption measurements are that the samples were heated to 150 ^{o}C and then degassed in a heating tank on a preprocessor under

vacuum at 150 °C for 12 h before testing.

3. The electrochemical tests and analysis of CO_2 reduction products.

The electrochemical tests were carried out on CHI 660E electrochemical workstation in a three-electrode system (at r.t.), in which the modified carbon cloth electrode (2 cm^2), saturated calomel electrode (SCE) and graphite electrode served as the working, reference and counter electrodes, respectively. The electrocatalytic CO_2RRs were conducted in an H-type cell. The cathode and anode compartments, which were separated by a Nafion 117 membrane, contain in each 25 mL 0.1 M $KHCO_3$ electrolyte. All potentials involved in this work were relative to reversible hydrogen electrode (RHE). The electrocatalyst ink was prepared by ultrasonically dispersing 5.0 mg electrocatalyst in the mixed solution containing 0.5 mL ethanol and 20.0 μL Nafion solution (5 wt.%). Then, 200.0 μL of electrocatalyst ink was dropped onto both sides of the carbon paper electrode and dried under an infrared lamp subsequently for electrocatalytic testing. The gas products were on-line quantitatively determined by gas chromatography equipped with a thermal conductivity detector (TCD; detecting H_2) and a flame ionization detector (FID; detecting CO and hydrocarbons). The liquid products were quantitatively analyzed by nuclear magnetic resonance (NMR) spectrometer (Bruker advance III 500 MHz). Firstly, 5 μL CH_3CN and 5 mL D_2O were mixed to prepare the mother liquor, then the mother liquor was diluted 20 times with D_2O to obtain a D_2O solution. After electrolysis, 400 μL cathode electrolyte was added to the NMR tube containing 100 μL of the above D_2O solution for NMR analysis. CH_3CN was used as the internal standard in NMR

analysis.

4. Figures

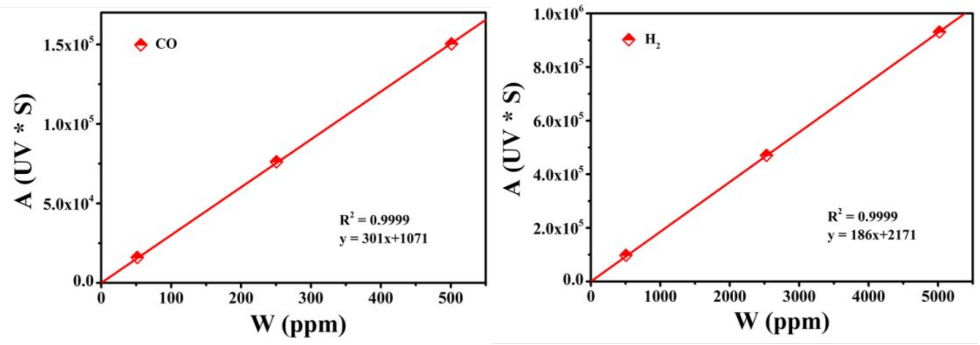

Fig. S1 Gas chromatography standard curves at different concentrations of CO **(a)** and H_2 **(b)**.

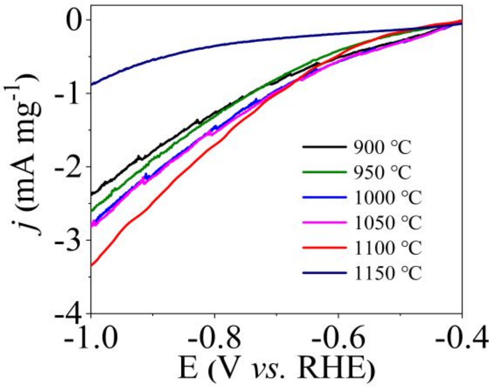

Fig. S2 LSV curves of CO_2RR on samples SeBN-C-***x*** (***x*** = 900, 950, 1000, 1050, 1100, and 1150 °C).

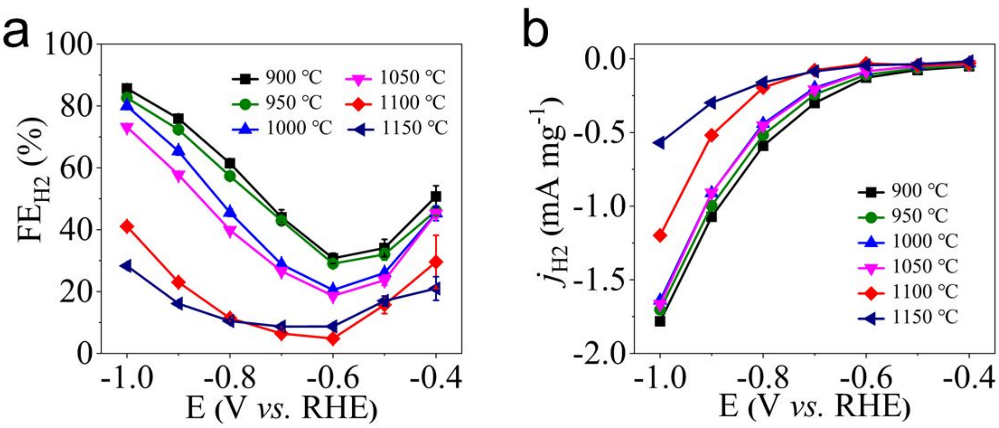

Fig. S3 FE$_{H2}$ **(a)** and j_{H2} **(b)** plots of CO_2RR at different potentials on samples SeBN-C-***x*** (***x*** = 900, 950, 1000, 1050, 1100, and 1150 °C).

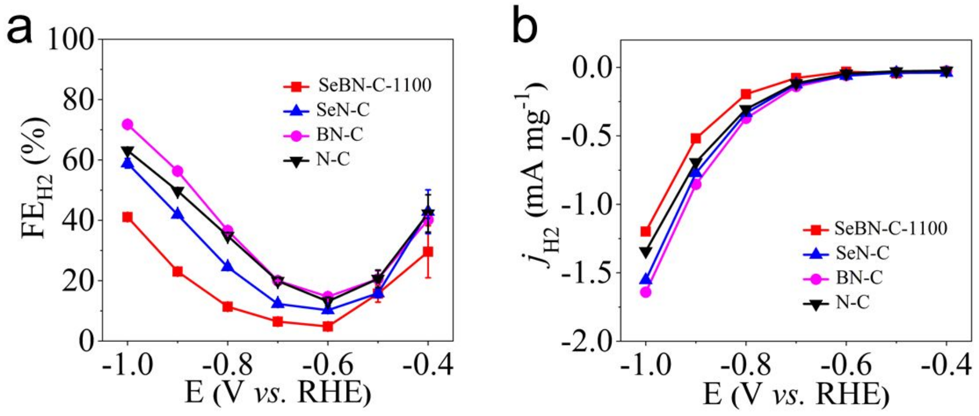

Fig. S4 FE$_{H2}$ **(a)** and j_{H2} **(b)** plots of CO_2RR on samples SeBN-C-1100, SeN-C, BN-C and N-C.

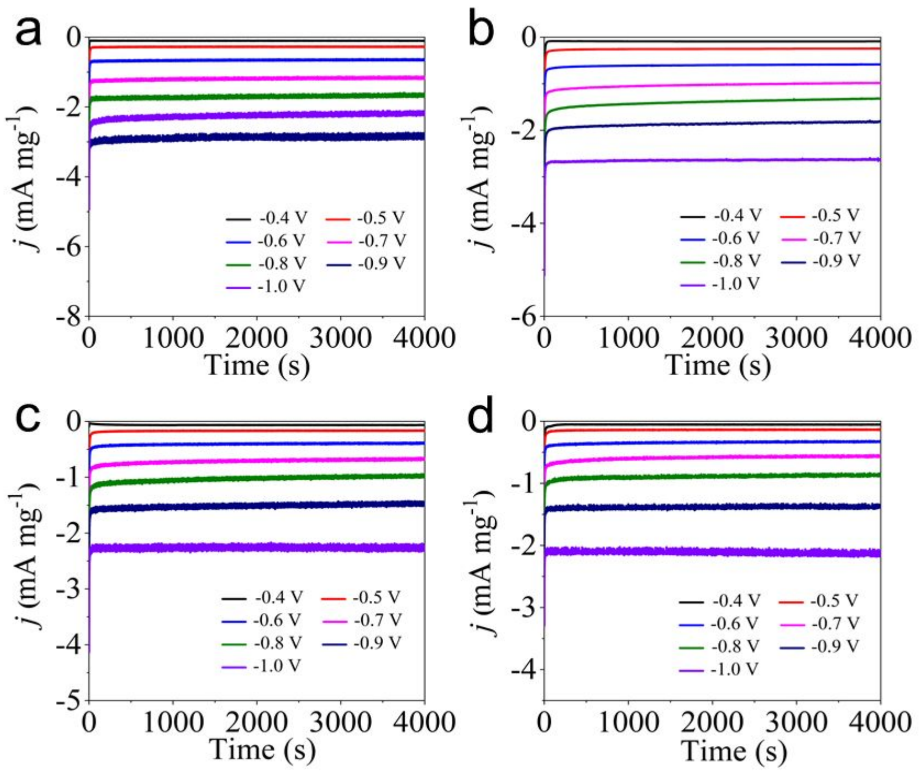

Fig. S5 Current density variation during CO_2RR in 0.1 M $KHCO_3$ aqueous solution of SeBN-C-1100 **(a)**, SeN-C **(b)**, BN-C **(c)** and N-C **(d)** at various potentials.

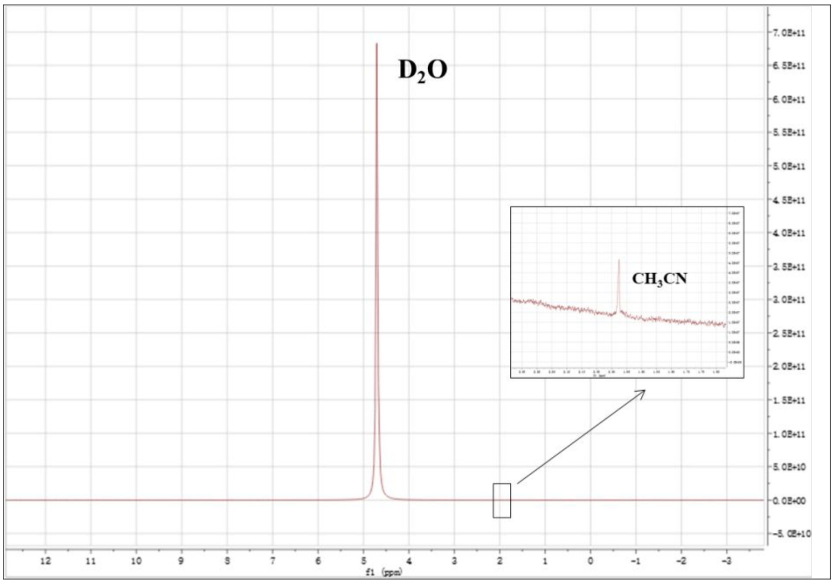

Fig. S6 Representative ^{1}H NMR spectrum of the electrolyte solution after CO_2 electrolysis operation.

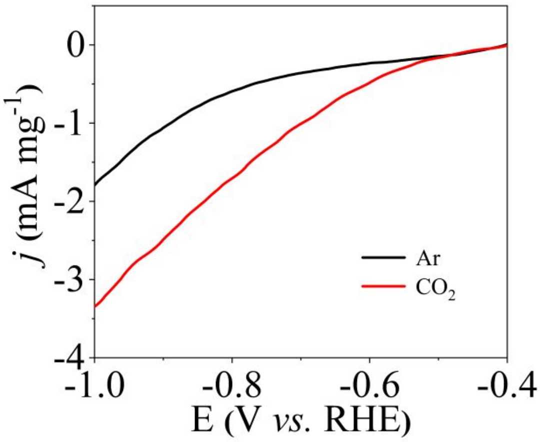

Fig. S7 LSV curves of the SeBN-C-1100 in Ar and CO_2-saturated 0.1 M $KHCO_3$ solution (scan rate: 50 mV s^{-1}).

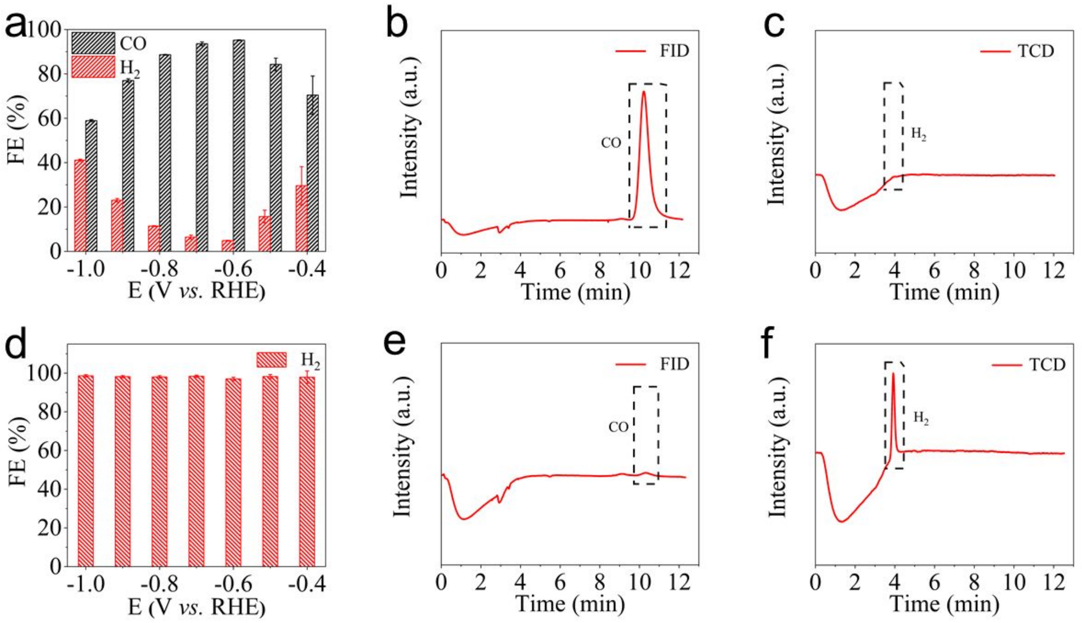

Fig. S8 FE_{H2} and FE_{CO} plots **(a),** gas chromatogram of the corresponding reduction product under the FID detector **(b)** and the TCD detector outcome **(c)** in CO_2-saturated 0.1 M $KHCO_3$ solution on the SeBN-C-1100 at different potentials**;** FE_{H2} plot **(d),** gas chromatogram of the corresponding reduction product under the FID detector **(e)** and the TCD detector outcome **(f)** in Ar-saturated 0.1 M $KHCO_3$ solution on the SeBN-C-1100 at different potentials.

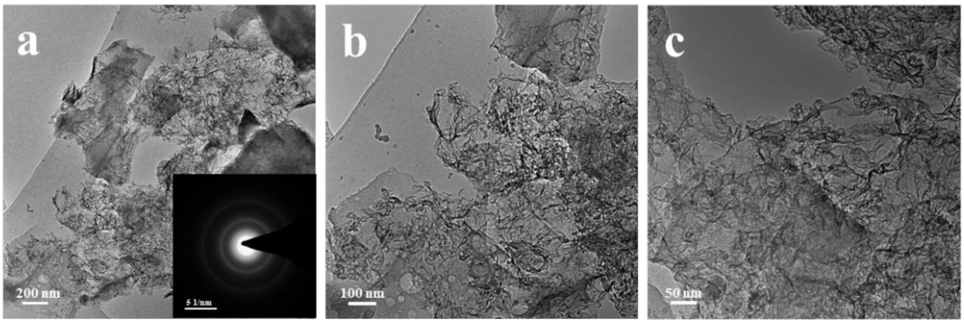

Fig. S9 Representative TEM images of SeN-C. The inset in **(a)** is the corresponding SAED pattern.

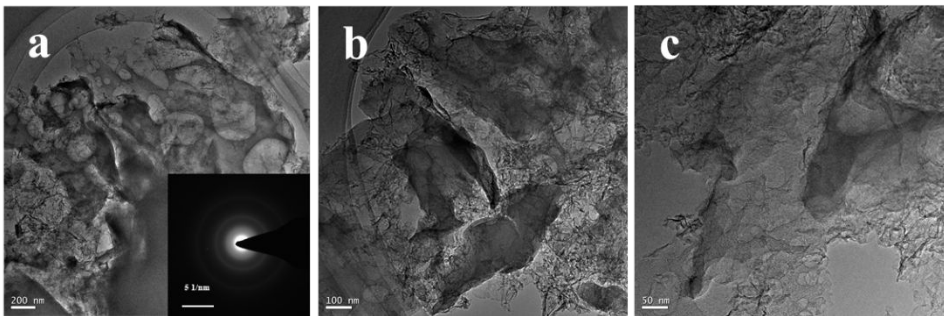

Fig. S10 Representative TEM images of BN-C. The inset in **(a)** is the corresponding SAED pattern.

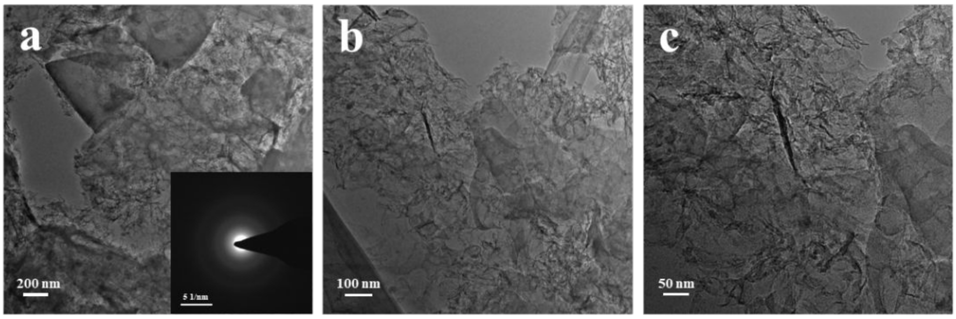

Fig. S11 Representative TEM images of N-C. The inset in **(a)** is the corresponding SAED pattern.

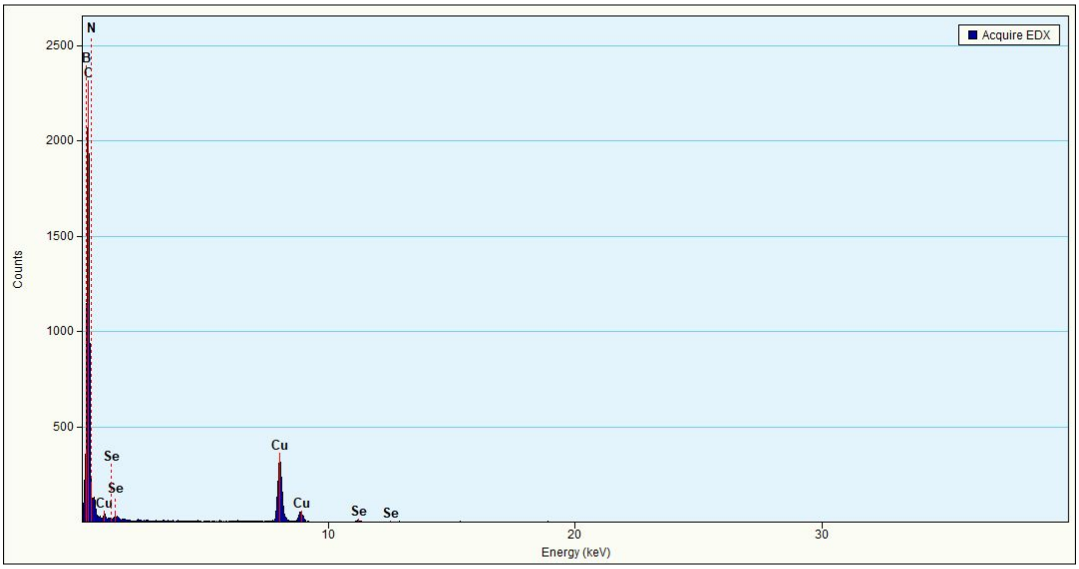

Fig. S12 EDX spectrum showing the presence of element in the sample SeBN-C-1100.

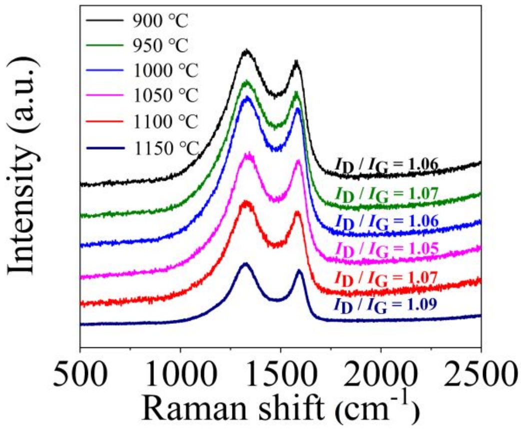

Fig. S13 Raman spectra of SeBN-C-***x*** (***x*** = 900, 950, 1000, 1050, 1100, and 1150 °C)

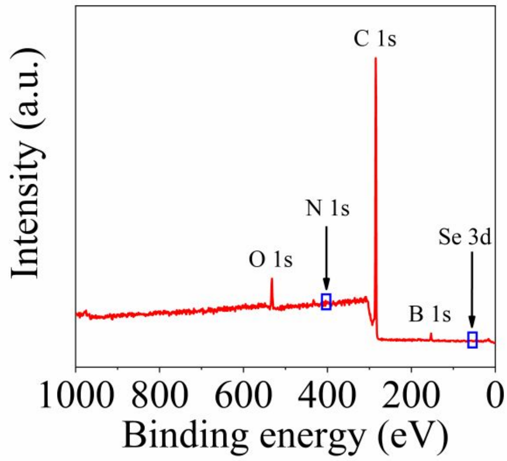

Fig. S14 XPS survey spectrum of the SeBN-C-1100.

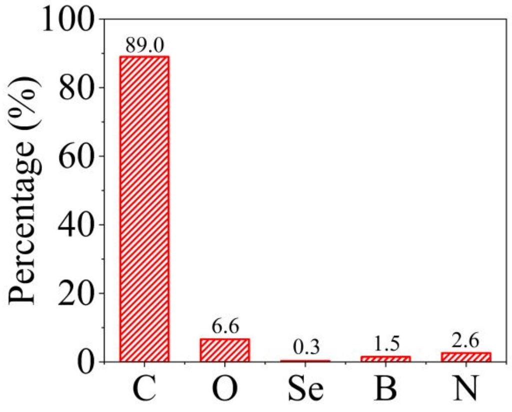

Fig. S15 Elemental content of SeBN-C-1100.

Tab. S1 Comparison of CO production on the SeBN-C-1100 in this work and electrocatalysts in previous reports.

**Electrocatalysts**	**Potential (V)**	**FE_{CO} (%)**	**Electrolytes**	**Ref.**
**SeBN-C-1100**	**-0.6 (*vs.*RHE)**	**95.2**	**0.1 M $KHCO_3$**	**This work**
Se-CNs	-0.6 (*vs.*RHE)	90.0	0.1 M $KHCO_3$	[S1]
2D-pg-C_3N_4	-1.1 (*vs.* Ag/AgCl)	~ 80.0	2.0 M $KHCO_3$	[S2]
PNC	-0.6 (*vs.*RHE)	74.0	0.1 M $KHCO_3$	[S3]
NP-C	-0.366 (*vs.*RHE)	60.0	0.1 M $KHCO_3$	[S4]
N-CNTs/SS-750	-1.1 (vs. Ag/AgCl).	75.0	0.1 M $KHCO_3$	[S5]
CF-120	-0.5 (*vs.*RHE)	~ 60.0	0.1 M $KHCO_3$	[S6]
NC(NH_3)	-0.5 (*vs.*RHE)	83.0	0.1 M $NaHCO_3$	[S7]

Supplementary references

[S1] Zhang, B.; Zhang, J.; Zhang, F.; Zheng, L.; Mo, G.; Han, B.; Yang, G. Selenium-doped Hierarchically Porous Carbon Nanosheets as an Efficient Metal-free Electrocatalyst for CO_2 Reduction. *Adv. Funct. Mater.* **2019**, *30*, 1906194.

[S2] Zhang, B.; Zhao, T. J.; Feng, W. J.; Liu, Y. X.; Wang, H. H.; Su, H.; Lv, L. B.; Li, X. H.; Chen, J. S. Polarized Few-layer g-C_3N_4 as Metal-free Electrocatalyst for Highly Efficient Reduction of CO_2. *Nano Res.* **2018**, *11*, 2450–2459.

[S3] Chen, K.; Deng, J.; Zhao, J.; Liu, X.; Imhanria, S.; Wang, W. Electrocatalytic Production of Tunable Syngas from CO_2 via a Metal-free Porous Nitrogen-doped Carbon. *Ind. Eng. Chem. Res.* **2021**, *60*, 7739–7745

[S4] Wang, W.; Borse, R. A.; Xie, J.; Wang, Y. Spontaneously Producing Syngas from MFC-MEC Coupling System Based on Biocompatible Bifunctional Metal-free Electrocatalyst. *Sci. China Mater.* **2021**, *64*, 592–600.

[S5] Liu, K. H.; Zhong, H. X.; Yang, X. Y.; Bao, D.; Meng, F. L.; Yan, J. M.; Zhang, X. B. Composition-tunable Synthesis of "clean" Syngas via a One-step Synthesis of Metal-free Pyridinic-N-enriched Self-supported CNTs: the Synergy of Electrocatalyst Pyrolysis Temperature and Potential. *Green Chem.* **2017**, *19*, 4284–4288.

[S6] Li, H.; Xiao, N.; Wang, Y.; Li, C.; Ye, X.; Guo, Z.; Pan, X.; Liu, C.; Bai, J.; Xiao, J.; Zhang, X.; Zhao, S.; Qiu, J. Nitrogen-doped Tubular Carbon Foam Electrodes for Efficient Electroreduction of CO_2 to Syngas with Potential-independent CO / H_2 Ratios. *J. Mater. Chem. A*. **2019**, *7*, 18852–18860.

[S7] Silva, W. O.; Silva, G. C.; Webster, R. F.; Benedetti, T. M.; Tilley, R. D.; Ticianelli, E. A. Electrochemical Reduction of CO_2 on Nitrogen-doped Carbon Catalysts with and Without Iron. *ChemElectroChem* **2019**, *6*, 4626–4636.
